# RETRACTED: Monda et al. Left Ventricular Non-Compaction in Children: Aetiology and Diagnostic Criteria. *Diagnostics* 2024, *14*, 115

**DOI:** 10.3390/diagnostics14232626

**Published:** 2024-11-22

**Authors:** Emanuele Monda, Gianantonio De Michele, Gaetano Diana, Federica Verrillo, Marta Rubino, Annapaola Cirillo, Adelaide Fusco, Federica Amodio, Martina Caiazza, Francesca Dongiglio, Giuseppe Palmiero, Pietro Buono, Maria Giovanna Russo, Giuseppe Limongelli

**Affiliations:** 1 Inherited and Rare Cardiovascular Diseases, Department of Translational Medical Sciences, University of Campania “Luigi Vanvitelli”, Monaldi Hospital, 80131 Naples, Italy; 2 Institute of Cardiovascular Science, University College London, London WC1N 3JH, UK; 3 Department of Maternal and Child Health, General Directorate for Health, 80131 Naples, Italy

The journal retracts the article, titled “Left Ventricular Non-Compaction in Children: Aetiology and Diagnostic Criteria” [1], cited above.

Following publication, concerns were brought to the attention of the Editorial Office regarding an overlap between this publication [1] and a previously published article [2] produced by a different authorship group.

Adhering to our complaints procedure, the Editorial Office and Editorial Board conducted an investigation that confirmed a significant overlap between the two articles [1,2], without appropriate acknowledgment or citation. As a result, the Editorial Board have decided to retract this paper as per MDPI’s retraction policy (https://www.mdpi.com/ethics#_bookmark30).

This retraction was approved by the Editor-in-Chief of the journal *Diagnostics*.

The authors did not provide a comment on this decision.
